# A Long-Term Risk? Prenatal POPs Exposure and Asthma in Young Adults

**DOI:** 10.1289/ehp.122-A28

**Published:** 2014-01-01

**Authors:** Lindsey Konkel

**Affiliations:** Lindsey Konkel is a Worcester, MA–based journalist who reports on science, health, and the environment. She is an editor for *Environmental Health News* and *The Daily Climate*.

Although previous research has suggested that prenatal exposures to persistent organic pollutants (POPs) may be harmful to a child’s developing immune system,^1^ few studies have investigated long-term outcomes in this regard. Findings reported in this issue of *EHP* provide evidence that exposure to certain POPs in the womb may be associated with an increased risk of developing asthma that persists into young adulthood.^2^

“The focus in immunotoxicity studies has often been on immunologic intermediates, such as immune cell counts. This study is unique in that it looks at a long-term clinically relevant outcome,” says Todd Jusko, an environmental epidemiologist at the University of Rochester, who was not involved in the study.

Researchers in Denmark assessed the relationship between asthma and prenatal exposure to polychlorinated biphenyls (PCBs), the pesticide hexachlorobenzene (HCB), and dichlorodiphenyldichloroethylene (DDE), a daughter compound of the pesticide DDT. PCBs, HCB, and DDT have been banned or restricted for many years due to concerns over human health effects, but they persist in the environment and the human body. Although levels of these chemicals have been dropping in the human population, diet—especially seafood consumption—remains an important route of exposure.^3^

Past studies have reported an association between prenatal exposure to DDE and asthma in children at ages 4^4^ and 7–10.^5^ Prenatal HCB exposure has been associated with airway hyperreactivity in rats.^6^ Other studies examining prenatal exposure to PCBs and asthma-like symptoms have yielded mixed results; two studies found an association with wheeze in infants^7^ and allergic sensitivity in children at age 7,^8^ while another reported an inverse association with allergic disease.^9^

**Figure:**
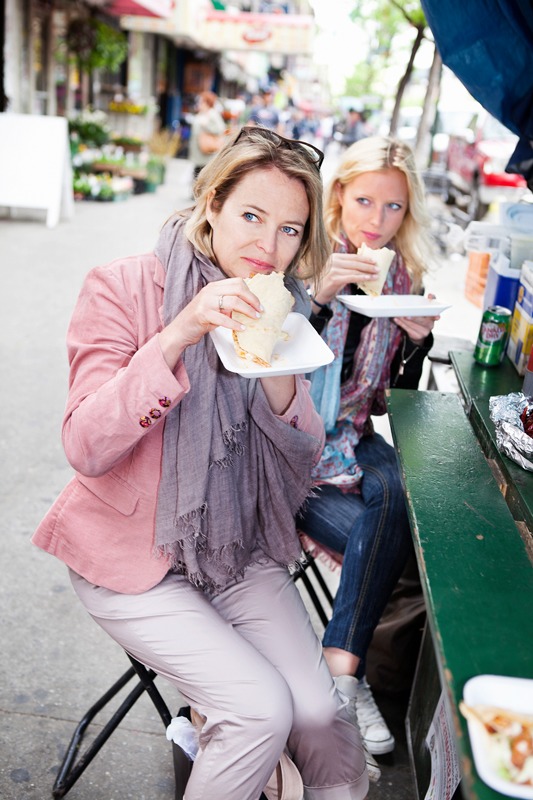
Children exposed to certain persistant organic pollutants in utero may be at increased risk for asthma that lasts into young adulthood. © Johner Images/Getty

Divergent findings may be due in part to the fact that previous studies have not followed up past childhood, say the authors of the new study. “A twenty-year follow-up allows us to distinguish between [true] asthma and asthma-like symptoms, such as wheeze, which may resolve in the first six to seven years of life,” says lead author Susanne Hansen, a doctoral candidate in epidemiology at the Statens Serum Institut in Copenhagen.

In the current study, the researchers assessed POP levels in blood samples from 872 pregnant Danish women. All samples were collected between 1988 and 1989, during the 30th week of pregnancy. Although blood levels of POPs in the cohort were higher than concentrations found in the general population today, they were average for women of childbearing age in Denmark in the 1980s, according to Hansen. The researchers used registry data and self-reports on asthma diagnosis and medication use since age 6 to identify asthma cases.

At age 20, children of mothers with the highest blood HCB concentrations during pregnancy were nearly twice as likely as children of mothers with the lowest concentrations to have been prescribed asthma medication. This is the first study to report an association between developmental HCB exposure and asthma in offspring, according to the researchers.

Similarly, children of mothers with the highest blood levels of one particular PCB congener, dioxin-like PCB-118, were nearly twice as likely as children of mothers with the lowest levels to have used asthma medications. The researchers also observed positive associations of HCB and PCBs with self-reported lifetime diagnosis of asthma, and between HCB and clinical diagnosis of asthma, although these associations were not statistically significant. None of the outcomes were associated with maternal DDE.

The mechanisms by which HCB and dioxin-like PCBs might influence asthma remain unclear. However, some researchers suspect PCBs and HCB interact with the aryl hydrocarbon receptor—a protein that, when activated in immune cells, may lead to a suppressed immune response.^10^

“The association between dioxin-like PCB 118 and asthma is an interesting one but not sufficient to suggest a general link between all dioxin-like chemicals and asthma,” says Michele La Merrill, a developmental toxicologist at the University of California, Davis. She says the possibility of such a general link is “a provocative question that would be worth continuing to ask in future studies.”

PCBs, HCB, and DDT have been shown to readily cross the placenta, making maternal blood levels during pregnancy a good proxy for fetal exposure. However, the current results are not sufficient for the researchers to make any claims about the relative importance of prenatal versus postnatal exposures. POPs can be transferred via breast milk, and the researchers had no data on breastfeeding habits for the mothers in their study. “Maternal serum levels could also be correlated with postnatal exposure,” Hansen says.

## References

[1] Glynn A Immune cell counts and risks of respiratory infections among infants exposed pre- and postnatally to organochlorine compounds: a prospective study. Environ Health 7 1 62 2008; 10.1186/1476-069X-7-62 19055819 PMC2637846

[2] Hansen S Maternal concentrations of persistent organochlorine pollutants and the risk of asthma in offspring: results from a prospective cohort with 20 years of follow-up. Environ Health Perspect 122 1 93 99 2014; 10.1289/ehp.1206397 PMC3888563 24162035

[3] Thompson MR Boekelheide K Multiple environmental chemical exposures to lead, mercury and polychlorinated biphenyls among childbearing-aged women (NHANES 1999–2004): body burden and risk factors. Environ Res 121 23 30 2013; 10.1016/j.envres.2012.10.005 23158727 PMC3578119

[4] Sunyer J Prenatal dichlorodiphenyldichloroethylene (DDE) and asthma in children. Environ Health Perspect 113 12 1787 1790 2005; 10.1289/ehp.8127 16330365 PMC1314922

[5] Karmaus W Infections and atopic disorders in childhood and organochlorine exposure. Arch Environ Health 56 6 485 492 2001; 10.1080/00039890109602896 11958547

[6] Michielsen C The environmental pollutant hexachlorobenzene causes eosinophilic and granulomatous inflammation and *in vitro* airways hyperreactivity in the Brown Norway rat. Arch Toxicol 76 2 236 247 2002; 10.1007/s00204-002-0326-x 12029387

[7] Stølevik SB Prenatal exposure to polychlorinated biphenyls and dioxins is associated with increased risk of wheeze and infections in infants. Food Chem Toxicol 49 8 1843 1848 2011; 10.1016/j.fct.2011.05.002 21571030

[8] Grandjean P Allergy and sensitization during childhood associated with prenatal and lactational exposure to marine pollutants. Environ Health Perspect 118 10 1429 1433 2010; 10.1289/ehp.1002289 20562055 PMC2957924

[9] Weisglas-Kuperus N Immunological effects of environmental exposure to polychlorinated biphenyls and dioxins in Dutch school children. Toxicol Lett 149 1–3 281 285 2004; 10.1016/j.toxlet.2003.12.039 15093274

[10] Benson JM Shepherd DM Dietary ligands of the aryl hydrocarbon receptor induce anti-inflammatory and immunoregulatory effects on murine dendritic cells. Toxicol Sci 124 2 327 338 2011; 10.1093/toxsci/kfr249 21948866 PMC3216417

